# Phenotypic variation of fruit and ecophysiological traits among maqui (*Aristotelia chilensis* [Molina] Stuntz) provenances established in a common garden

**DOI:** 10.1038/s41598-021-04013-0

**Published:** 2022-01-07

**Authors:** Marco A. Yáñez, Benita González, Sergio E. Espinoza, Hermine Vogel, Ursula Doll

**Affiliations:** 1 Instituto de Investigación Interdisciplinaria, Vicerrectoria Académica, Universidad de Talca, 2 Norte 685, P.O. Box 747, Talca, Chile; 2 Centro de Plantas Nativas de Chile (CENATIV), Facultad de Ciencias Agrarias, Universidad de Talca, 2 Norte 685, P.O. Box 747, Talca, Chile; 3 Facultad de Ciencias Agrarias y Forestales, Universidad Católica del Maule, Avenida San Miguel 3605, Talca, Chile

**Keywords:** Ecology, Plant sciences

## Abstract

The domestication of forest species has traditionally relied on productivity issues. However, today there are concerns about the potential responses of natural populations and new cultivars to extreme climatic conditions derived from climate change and how to incorporate this knowledge into the domestication programs. *Aristotelia chilensis* (Molina) Stuntz (‘Maqui’) is a widely distributed native species in Chile. Its berry is considered a “super fruit” with an increasing interest in the food industry. This study investigated the phenotypic variation of growth, fruit, and ecophysiological traits of 20 *A. chilensis* clones originated from six provenances along the latitudinal gradient and established in a common-garden experiment in the Mediterranean zone of central Chile (center part of the species distribution). Differences among provenances were observed for most of the  traits under study, especially between the northern and southernmost provenances (i.e., San Fernando versus Entre Lagos). Northern provenances showed higher development of vegetative tissue and fruit yield but lower intrinsic water use efficiency (WUE_int_) compared with southern ones. Clonal variation within provenances was found significant for the ripening index, WUE_int_, and fruit number and weight but not significant for traits related to the crown and leaf morphology. A genetic differentiation due to latitudinal cline was not evident in this study, but differences among provenances suggest local adaptation for some traits. The genotypic variation in productive traits must be considered in the outgoing domestication of the species and future selection programs.

## Introduction

The global consumption of fruits is increasing due to the higher awareness of their health benefits and nutritional values^1^. Forests have an essential role in future food security as they represent a valuable source of vital nutrients and natural antioxidants^2,3^. Among fruits, berries have an enormous potential for the food industry as they are a rich source of bioactive compounds such as phenolic acids and anthocyanins^4^. Nonetheless, there are concerns about how the increasing temperatures and droughts due to climate change might affect the natural populations of important fruit plant species contributing to food security.

*Aristotelia chilensis* (Molina) Stuntz, commonly named “Maqui”, is in Chile a native species whose range of distribution spans over 15° latitude (c.a. 1700 km) from Mediterranean semi-arid to temperate sub-humid and humid climates^5,6^. Its 6-mm red/purple colored berries are considered a “super fruit” because of their great health properties^7,8^. Its soluble solids are higher than in other berries such as blueberries (*Vaccinium corymbosum)*, pomegranates (*Punica granatum*), blackberries (*Rubus fruticosus*), raspberries (*Rubus idaeus*), and cranberries (*Vaccinium oxycoccos*)^9^. They also have the highest antioxidant content among the fruits currently traded worldwide^5^. Recent studies have demonstrated its powerful antioxidant, cardioprotective, anti-inflammatory, gastroprotective, antidiabetic, and antibacterial capacity^4,9–15^. Currently, Maqui berries are wildcrafted directly from the natural populations, which contributes to the degradation and loss of genetic diversity^16^. To sustain the industry's growing demand, domestication studies of the species have focused on genetic selection and adaptation to cultural management techniques. However, there is little knowledge about the phenotypic and genetic variation of fruit and ecophysiological traits of natural maqui populations, information that might guide the future breeding and selection of outstanding genotypes.

One of the most important approaches to understanding the phenotypic variation and adaptation to local conditions is the use of common garden experiments^17,18^. Studies suggest that some species covering large geographical and environmental gradients may develop local adaptation and phenotypic clines in growth and functional traits^17,19^, but this ability may change by species^20^. For instance, some forest species exhibit a higher genetic differentiation in growth and phenological traits than others describing wood anatomy or leaf physiology across their natural distribution^17,19^, whereas others exhibit genetic differentiation for physiological traits such as photosynthetic capacity and leaf water status^21,22^. Moreover, studies in common garden experiments have reported high provenance differentiation in the fruit and leave morphology of fruit species^23–25^, and in the accumulation of bioactive compounds such as polyphenols and anthocyanins in berries species^26–28^.

Some studies suggest a potential genetic variation that may be used in the domestication process of *A. chilensis*. In 1-year old clonal plants, Vogel et al.^16^ found differences in growth, leaf morphology, and fruit set traits among provenances that originated across the latitudinal gradient of the species. In a nursery experiment, Moya et al.^29^ also reported differences in these traits at the clone level, although this study considered only three commercial varieties. Otherwise, there are only a few studies addressing the genetic diversity and structure of *A. chilensis*. Cona et al.^30^ found a high genetic diversity in the species in a latitudinal range of 15 degrees, which following Hamrick^31^, is expected for a non-domesticated species with anemophily and entomophile pollination. Salgado et al.^32^ found that most of the genetic variation, evaluated by AFLP and chloroplast microsatellites, was explained by the differences within provenances (i.e., clonal variation) than among provenances. These authors point out the existence of little genetic structure and relatively high gene flow between populations. However, it is known that although these types of molecular techniques grasp well the neutral genetic variation, they have a lower performance on the variation of adaptive traits^33,34^. Thus, the extent of variation of adaptive traits of the species at the clonal and population level still remains unknown, and little is known in broadly distributed species such as *A. chilensis*, especially in the southern hemisphere. Regarding that climate conditions vary across the latitudinal gradient of the species distribution, we explored the question of What is the extent of genetic differentiation of fruit and ecophysiological traits between and within provenances of *A. chilensis*? The present study investigated the phenotypic variation of fruit, ecophysiological traits, and leaf traits of 20 *A*. *chilensis* clones from six provenances exhibiting latitudinal-related climate differences in Central Chile, and assessed in a 7-years old common-garden experiment.

## Methods

### Study site, plant material, and experimental design

A common garden experiment was established at the Experimental Station of Universidad de Talca in Panguilemo, Maule Region, Chile (latitude 35° 21′ 53.5″, longitude 71° 35′ 58.5″, altitude of 120 m a.s.l.). This location is nearly the center of the latitudinal distribution of the species. The site is characterized by a Mediterranean climate, with a mean annual temperature of 14.7 °C and precipitation of 758 mm. The soil is classified as San Rafael soil series (alfisols), with sandy clay loam texture, moderate drainage, and 50–90 cm deep. Soil nutrient analysis showed a content of N = 8 mg kg^−1^, P = 47 mg kg^−1^, K = 209 mg kg^−1^, Mn = 42.8 mg kg^−1^, Zn = 12.3 mg kg^−1^, Cu = 3.9 mg kg^−1^, Fe = 51.7 mg kg^−1^, B = 0.87 mg kg^−1^, pH = 6.1, and organic matter = 1.1%. Before planting, the soil was plowed to a depth of 40 cm, harrowed, and mounding of 40 cm height. On the planting rows, weeds were controlled by mulching and mechanically and between rows by mowing. A drip irrigation system watered the plants during the growing seasons, from October to March.

**Figure 1:**
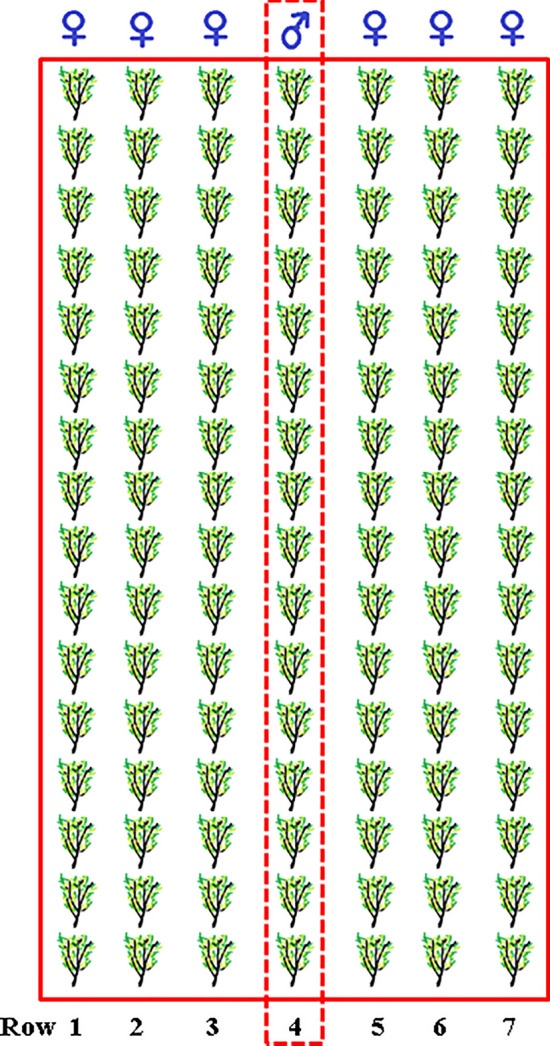
View of the field trial scheme.

In May 2012, 45 female *A. chilensis* clones from eight wild provenances covering an area over 750 km or a latitudinal range of six degrees were arranged in a randomized design with 4–5 replicates. The experimental unit was a single-tree plot with 3.5 m between and 1.2 m within rows. For optimal free pollination, five male clones were located in the central row within the trial (Fig. 1). The planting material consisted of 30-cm-long rooted cuttings. During the seventh growing season (2018–2019), 20 of the most outstanding fruit producers and well-adapted clones were selected for this study, which belonged to six provenances (hereinafter, SanFer, Romeral, and Talca named as northern provenances, whereas Mulchen, Pucvilla, and Enlagos named as southern provenances). The number of clones ranged from two to five by provenance. Table 1 shows the location and climatic data for the selected provenances (Climate data sources were www.es.climate-data.org, www.minenergia.cl/exploradorsolar/), and the number of clones per each provenance. The experiment was performed in accordance with relevant guidelines and regulations.

**Table 1:** Location, altitude, Köppen climatic classification, climatic data and solar radiation for the *Aristotelia chilensis* (Maqui) provenances under study.

Provenance	Region	Latitude (°S)	Longitude (°W)	Altitude (m)	Köppen clasification^1^	Mean temperature (°C)	Temperature Min–Max^2^ (°C)	Precipitation (mm)	Global radiation (MJ m^−^^2^)	Number of clones	De Martonne aridity index
San Fernando (SanFer)	Northern	34° 41′	70° 50′	530	Csc	14.0	7.0–20.9	552	19.1	5	23
Romeral (Romer)		34° 57′	70° 57′	495	Csb	13.5	6.4–20.6	833	18.5	3	36
Talca		35° 34′	71° 22′	275	Csb	13.9	6.7–21.1	869	18.1	3	36
Mulchen	Southern	37° 40′	71° 01′	329	Csb	13.2	6.7–19.6	1306	17.6	2	56
Villarrica (Pucvilla)		39° 16′	71° 59′	190	Csb	10.8	5.1–16.6	2265	15.6	2	109
Entre Lagos (Enlagos)		40° 40′	72° 33′	165	Cfb	11.4	6.5–16.4	1855	13.7	5	87

^1^Köepen climate classification, Csc: Temperate Mediterranean climate (mild summer), Csb: Temperate Mediterranean climate (warm summer), Cfb: Temperate Marine West Coast climate (warm summer).

^2^Min and Max correspond to Minimum and maximum temperatures. The De Martonne aridity index was estimated as MAP/(MAT + 10). The higher the index, the lower the aridity.

### Leaf-level physiological traits

Leaf-level physiological measurements were taken nine times during the study period (from December 7, 2018 to March 28, 2019) at each plant per clone. Net saturated photosynthesis rate (*A*_*sat*_, μmol m^−2^ s^−1^), stomatal conductance (*g*_*s*_, mmol m^−2^ s^−1^) and intrinsic water use efficiency (WUE_int_, μmol mmol^−1^) were measured on one fully expanded leaf in the upper third of the plant, using a portable gas exchange system LI 6800 (LICOR Inc., Lincoln, NE, USA). Initial chamber conditions were set up close to the ambient conditions during measurements, with a temperature of 20 °C, CO_2_ concentration of 400 ppm, relative humidity of 50%, and PAR of 1800 µmol m^−2^ s^−1^. Measurements were performed between 09.00 and 12.00 h at local time.

### Fruit ripening index

The ripening stage of *A*. *chilensis* berries was assessed on five dates from November 25 to December 29, 2018. According to the fruit color, we defined three ripening stages: green (berry development), reddish (beginning of ripening), and black purple (end of ripening). A representative branch of each plant was selected, and the percentage of fruit in each category was visually evaluated. Then, we elaborated a ripening index representing a weighted average at the plant level: Ripening index = (1 × Percentage green fruit + 2 × Percentage of reddish fruit + 3 × Percentage of black-purple fruit) 100^–1^. The index varies between 1 (all fruit in green color) and 3 (all fruit in black purple color).

### Leaf morphological traits

At the beginning of summer, 20 leaves per plant were randomly collected in the upper half of the crown for leaf morphological assessments. First, we measured the ratio between the wide of the curved leaf relative to the wide when it is flattened, from now on denominated leaf curve ratio. The same foliage was used to estimate the lamina area (LA) using the formula by Vogel et al.^16^: LA = [(L_l_ − L_w_/2)L_w_/2 + п/2(L_w_/2)^2^], where L_l_ is lamina length and L_w_ is lamina width. Then, the leaves were oven-dried at 65 °C to calculate the specific leaf area (SLA) as the leaf area to dry weight ratio.

### Fruit productivity and quality

Similarly, at the end of ripening, four twigs were randomly collected at each plant to measure the growth of the previous and current growing season (2017–2018). The foliage and fruit were detached from the twigs and stored in plastic bags for further analysis. The leaves were scanned for leaf area (FA) determination using a bed scanner and the software Image J (developed by the National Institute of Health). The fruit on each twig was counted and weighted in fresh (FFW). Then, it was oven-dried at 65 °C for 48 h for dry mass determination. Additionally, we calculated a productivity index as the FFW to FA ratio. Twigs were also measured in the basal diameter and length using a digital caliper and a metric tape, respectively.

Additionally, 60 g of fruit was collected from each plant to assess fruit quality. In a subsample of 20 randomly collected berries, fruit diameter was determined with a digital caliper. The remaining fruit was put in plastic bags and sent to the laboratory for polyphenols and anthocyanins analysis according to Folin-Ciocalteu and differential pH colorimetric methods described by Singleton and Rossi^35^ and Giusti and Wrolstad^36^, with some modifications as described by González et al.^37^.

### Crown measurements

From each plant, two photos were taken perpendicular to the planting row with white cloth behind as a high contrast backdrop using a 28–70 mm 1:2.8–4.0 D zoom lens (Sigma Corp., Kanagawa, Japan) mounted on a D70 digital SLR (Nikon Corp., Tokyo, Japan). The plant height was measured with a graduated pole. Then, images were processed with the software UrbanCrowns for Windows (USDA, Forest Service) to determine the crown volume and density^38^.

### Statistical analysis

First, to assess the interaction between provenances and measured dates within the growing season for the ripening index and leaf-physiological parameters, the following linear mixed model was considered:

1 $${y}_{ijkl}=\mu +{P}_{i}+{c(P)}_{ij}+{Date}_{k}{+ P\times Date}_{ik}+{c(P)\times Date}_{ijk}+ {\varepsilon }_{ijkl}$$

where ${y}_{ijkl}$ is the phenotypic observation; $\mu$ is the overall mean; ${P}_{i}$ is the fixed effect of provenance i; ${c}_{j}$ is the random effect of clone j within provenance i; ${Date}_{k}$ is the fixed effect of date k; ${P\times Date}_{ik}$ and ${c(P)\times Date}_{ijk}$ are interactions among factors; and ${\varepsilon }_{ijkl}$ is the random residual effect. The variance–covariance matrix of residual effects repeated over time was modelled to account for the temporal variation. Analysis of the Akaike (AIC) and Bayesian (BIC) information criteria (data not included) showed the best fit for the unstructured (UN) structure compared with the compound symmetry (CS). Second, a reduced model was used for leaf morphological, crown, and fruit and traits, which were measured once during the growing season.

2 $${y}_{ijk}=\mu +{P}_{i}+{c(P)}_{ij}+{\varepsilon }_{ijk}$$

where ${y}_{ijkl}$ is the phenotypic observation; $\mu$ is the overall mean; ${P}_{i}$ is the fixed effect of provenance i; ${c}_{j}$ is the random effect of clone j within provenance i; and ${\varepsilon }_{ijk}$ is the random residual effect. The models were fitted using Proc Mixed in SAS 9.4 (SAS Institute Inc., Cary, NC). Count data such as fruit number was modeled as a generalized lineal mixed model, using the log-link function for Poisson distribution. Mean comparisons for fixed effects were made using the Tukey-multiple comparison test. For random effects, adjusted means using E-BLUE (empirical best linear unbiased estimation) were calculated as described in Welham. et al.^39^. A multivariate cluster analysis based on FFW, twigs growth, FA, and WUE_int_ was carried out on the clones using the Ward’s method in the software JMP GENOMICS 7.1. Because of the low replication of the experiment, the statistical significance of the analyses was considered at a probability level of 0.1.

## Results

### Variability among genotypes of the ripening index and leaf-level physiology

**Figure 2:**
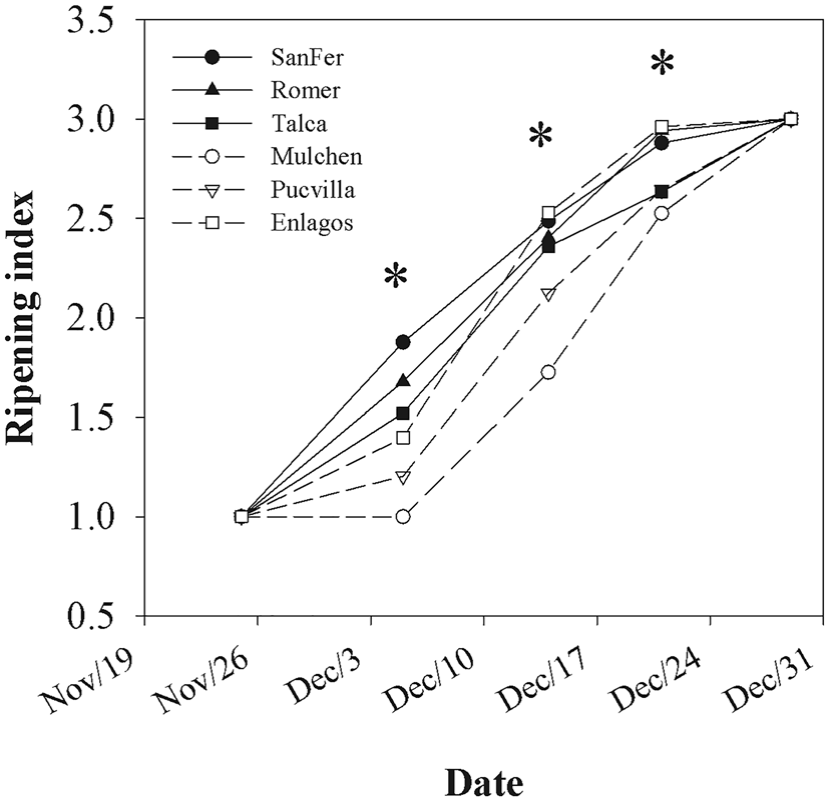
Mean ripening index for *A. chilensis* provenances and measurement date. *Significant differences at a probability level of 0.1.

**Figure 3:**
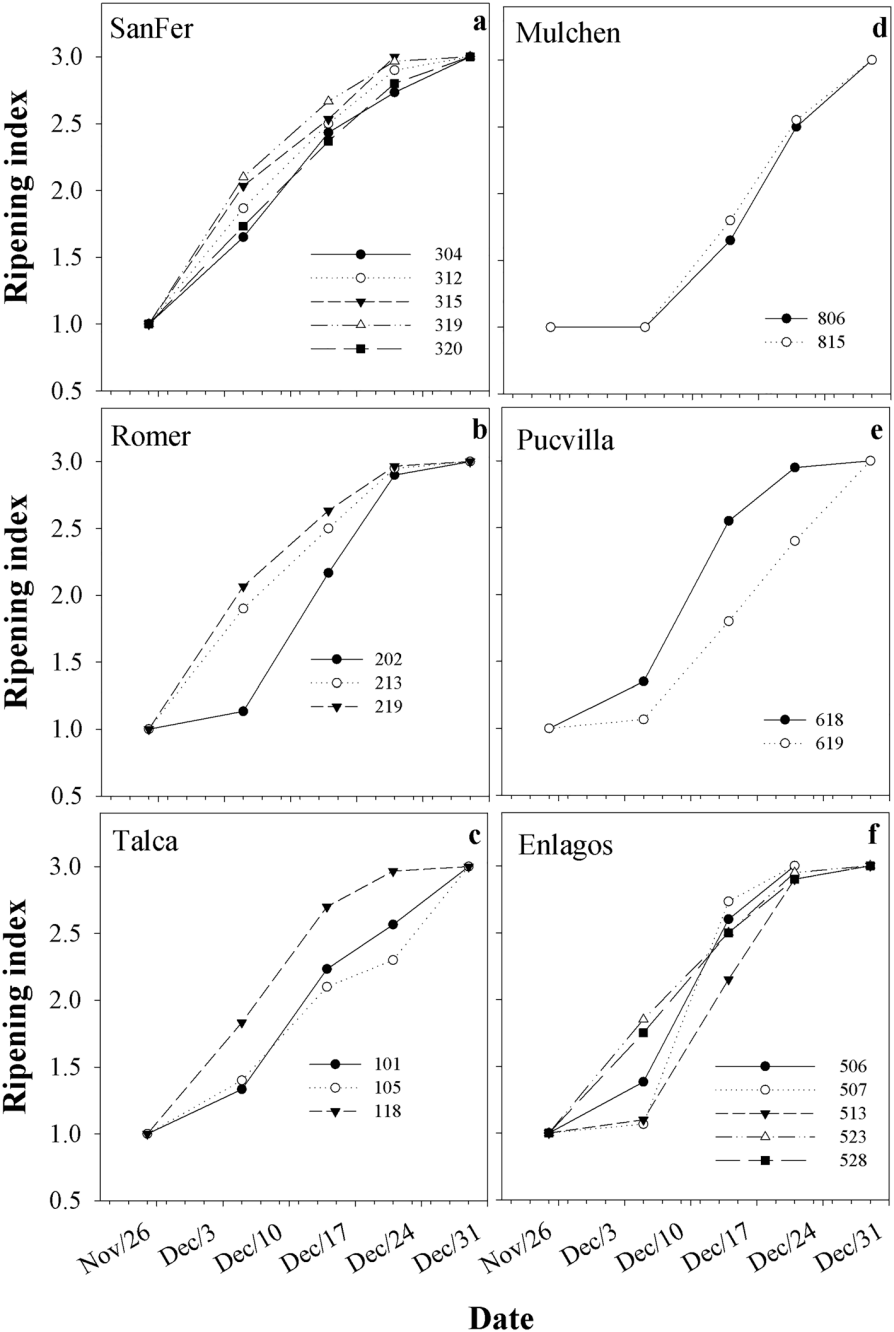
Adjusted means (based on E-BLUP) of the ripening index for *A. chilensis* clones and measurement date. *Significant differences at a probability level of 0.1.

In all studied provenances, the ripening period, from the green to the black-purple stage, lasted approximately one month, although provenances varied in the ripening index by date (*P* = 0.0075, Table 2, Fig. 2). No latitudinal pattern associated with the provenance origin was observed on fruit maturation (i.e., clinal genetic variation). Fruit from the northern provenances of SanFer and Romer and the southernmost Enlagos ended ripening earlier than the others (Fig. 2). Differences in the ripening index among provenances were observed since the second measurement date, being the Mulchen provenance the one with the most delayed ripening. There was also a significant clonal variation within provenance on the ripening index (*P* = 0.0850, Table 2), represented in Fig. 3. As can be seen, excepting SanFer and Mulchen, the provenances exhibited some clonal variation on the starting and end of ripening.

**Table 2:** *P*-values for fixed and random effects from the analysis of variance conducted on *Aristotelia chilensis* (Maqui) for leaf physiology (light-saturated photosynthetic rate (*A*_*sat*_), stomatal conductance (*g*_*s*_), transpiration (*E*), intrinsic water use efficiency (WUE_int_), instantaneous water use efficiency (WUE_ins_)).

Effect	Leaf physiology				
	Ripening index	*A*_*sat*_	*g*_*s*_	*E*	WUE_int_
Provenance (P)	**0.0159**	0.1182	**0.001**	**0.001**	**0.0007**
Date	**< 0.0001**	**< 0.0001**	**< 0.0001**	**< 0.0001**	**< 0.0001**
Proven × date	**0.0075**	**0.0023**	0.3081	0.3578	0.9086
Clone (P)	**0.0850**	**0.0748**	0.4676	0.9522	0.3122
Clone (P) × date	0.9621	0.8891	**0.0411**	0.9501	**0.0096**

Significant effects (*P*-value < 0.1) are shown in bold type. The effects are provenance (Proven), Date and Clone.

**Figure 4:**
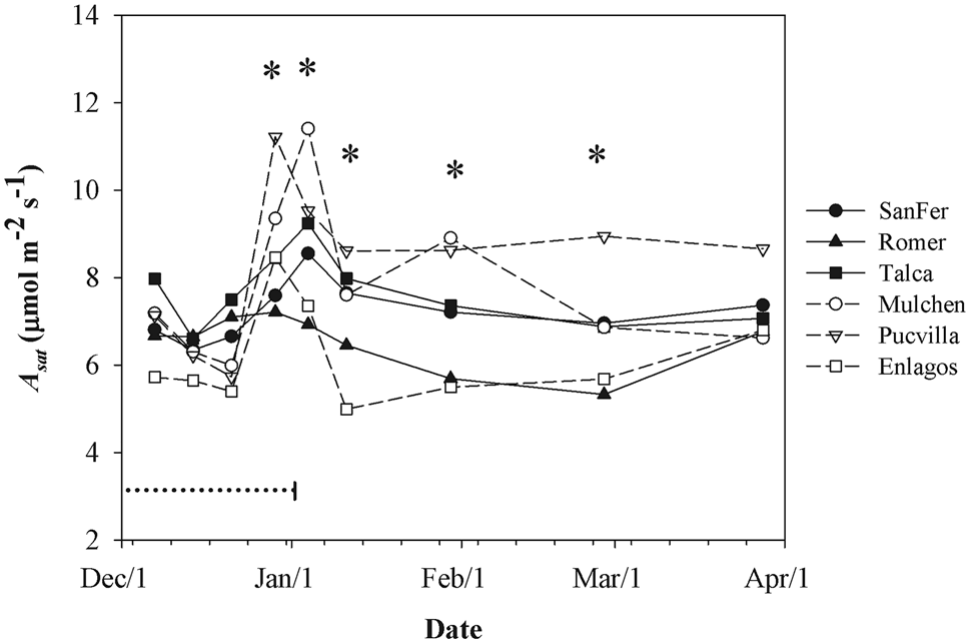
Means light-saturated photosynthetic rate (*A*_*sat*_) for *A. chilensis* provenances and measurement date. *Significant differences at a probability level of 0.1. Horizontal dotted line represents the ripening period.

**Figure 5:**
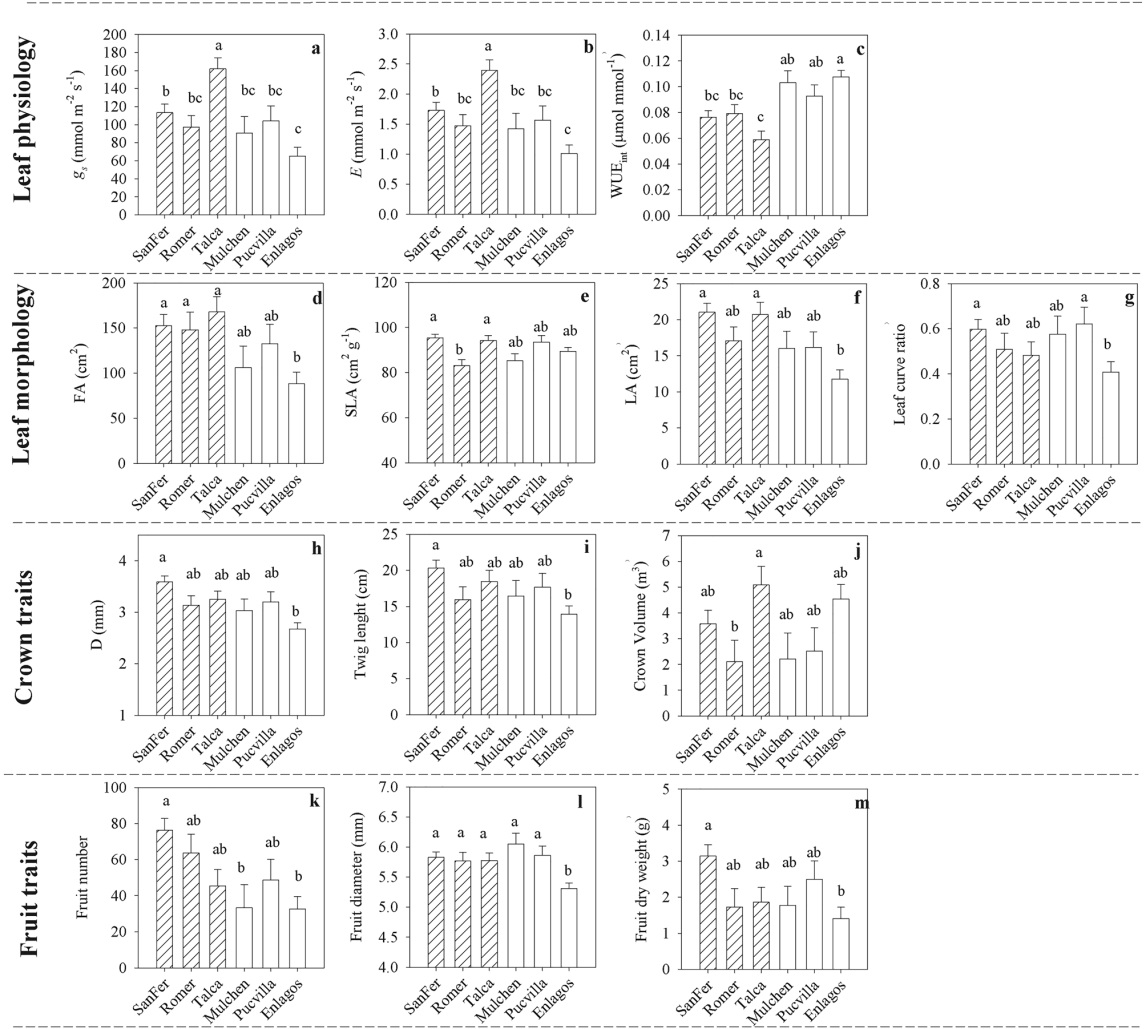
Means for leaf-physiological, morphological, crown, and fruit traits of *A. chilensis* provenances. Different letters indicate significant differences among provenances, at a probability level of 0.1 in the Tukey’s comparison test. Dashed and white bars correspond to northern and southern provenance origin, respectively.

Except for *A*_*sat*_, leaf-level physiological traits differed among provenances (*P* < 0.001, Table 2), but no clinal variation was observed (Fig. 5a–c). Differences in *A*_*sat*_ among provenances varied by date, ranging between 5.0 and 11.5 µmol m^−2^ s^−1^ (Fig. 4). These differences among provenances were expressed mainly after the end of ripening. During the whole study period, the provenances of Mulchen and Pucvilla had the highest and Romer and Enlagos the lowest *A*_*sat*_ values. Provenances significantly differed in *g*_*s*_, *E*, and WUE_int_, ranging from 65 to 162 mmol m^−2^ s^−1^, 1.0–2.4 mmol m^−2^ s^−1^, and 0.059–0.107 µmol mmol^−1^, respectively (Table 2, Fig. 5a–c). The local provenance Talca and the southernmost provenance Enlagos had the highest and lowest values for *g*_*s*_ and *E*, respectively. The highest stomatal conductance of the provenance Talca matched with the lowest WUE_int_ relative to the other provenances (Fig. 5c).

**Figure 6:**
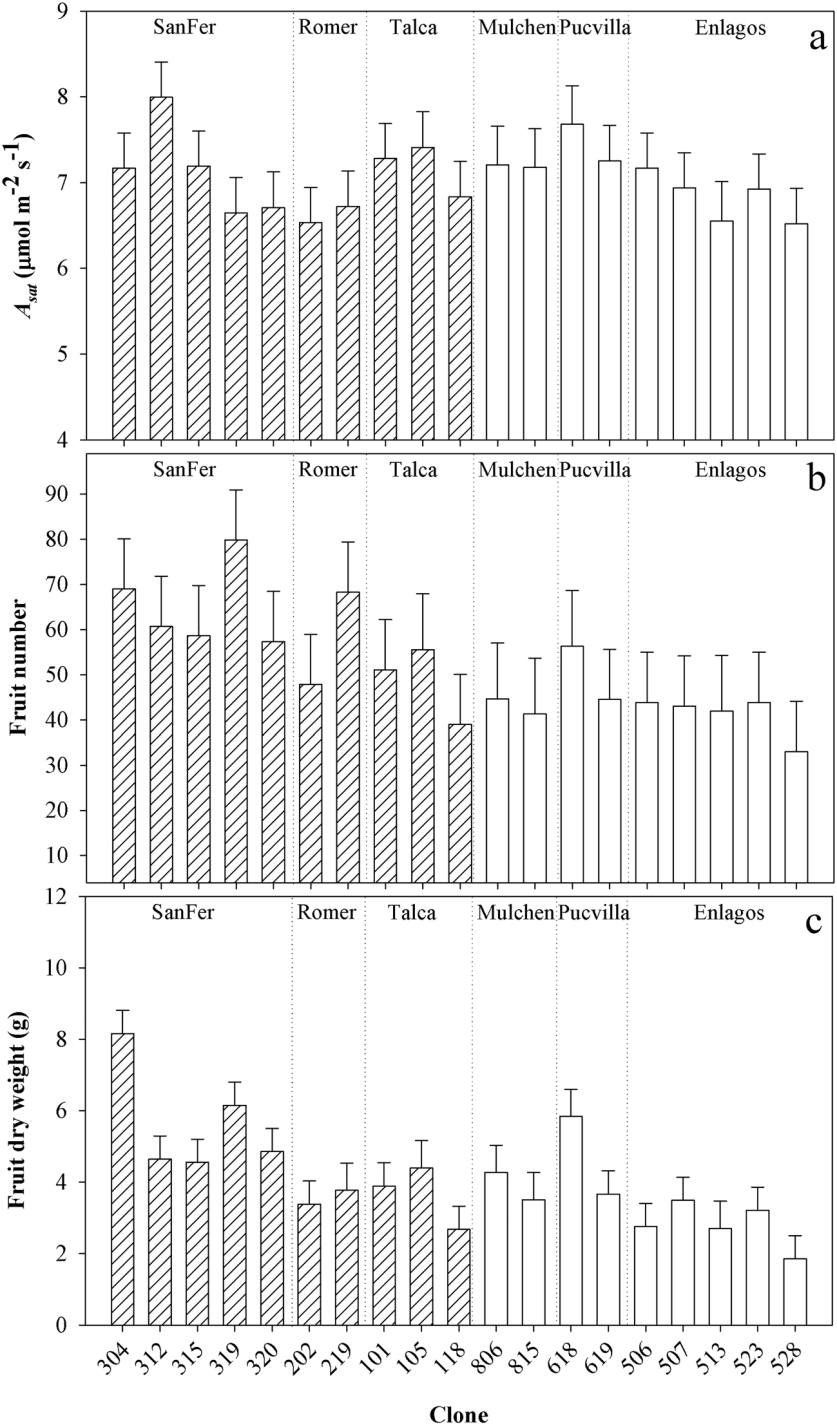
Adjusted means (based on E-BLUP) for light-saturated photosynthetic rate (*A*_*sat*_) (**a**), fruit number (**b**), and fruit dry weight for *A. chilensis* clones. Dashed and white bars correspond to northern and southern provenance origin, respectively.

**Figure 7:**
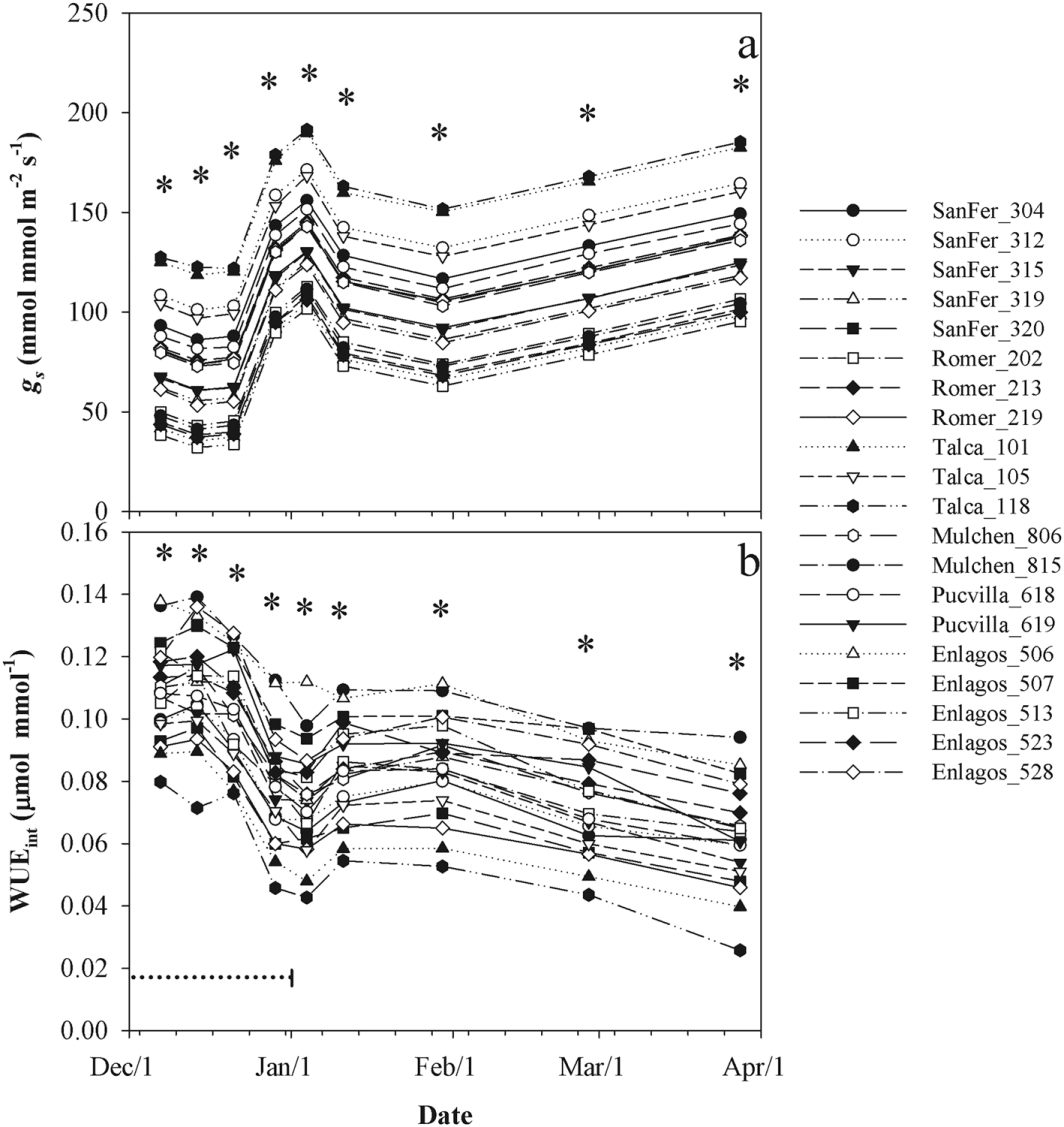
Adjusted means (based on E-BLUP) of stomatal conductance (*g*_*s*_), and intrinsic water use efficiency (WUE_int_) for *A.chilensis* clones and measurement date. *Significant differences at a probability level of 0.1. Horizontal dotted line represents the ripening period.

There was also a significant clonal variation within provenances on *A*_*sat*_ (*P* = 0.0748), likely explained by the greater clonal differences in provenance SanFer (Table 2, Fig. 6a). Moreover, clonal differences on *g*_*s*_ and WUE_int_ varied by date and were significant across all the study periods, but the differences among clones and the relative position between them were more consistent for *g*_*s*_ than WUE_int_ (Table 2, Fig. 7). In the end, clones with higher and lower *g*_*s*_ differed 2-fold, while for WUE_int_ 3.7-fold. High values for WUE_int_ were observed during the ripening period. Overall, southern provenances (Mulchen, Pucvilla, Enlagos) tended to have higher WUE_int_ than northern provenances (SanFer, Romer, Talca) (Fig. 7).

### Variability among genotypes on leaf morphology and crown characteristics

Most leaf morphological and crown traits differed significantly among provenances, but not at the clone level (Table 3). The northern provenance of SanFer, Romer, and Talca developed more foliage (FA and LA, Fig. 5d,f) than the southernmost provenance Enlagos. Growth (twig diameter and length, Fig. 5h,i) was also higher in the northernmost provenance SanFer than the southernmost provenance Enlagos. Similarly, provenances SanFer and Pucvilla significantly expressed less leaf curvature than the provenance Enlagos (Fig. 5g). However, despite the fact that *A*. *chilensis* is widely distributed over contrasting environmental conditions, no clinal genetic variation was attributed to these types of traits. For instance, northern provenances Romer and Talca also had a similarly high value of LA, but they significantly differed in crown volume and SLA (Fig. 5e,j). Southern provenances Mulchen and Pucvilla had intermediate values for twig growth and leaf morphology (Fig. 5). There were no significant differences among provenances for the FFW to FA ratio, crown density, and tree height, with general means of 0.035 g cm^−2^, 64.8%, and 2.3 m, respectively (Table 3).

**Table 3:** *P*-values for fixed and random effects from the analysis of variance conducted on *Aristotelia chilensis* (Maqui) for leaf morphology (foliage area (FA), specific leaf area (SLA), leaf area (LA), Leaf curvature (leaf curve), fruit fresh weight (FFW) to foliage area ratio), crown traits (basal twig diameter (D), twig length, crown volume, crown density, tree height (H)) and fruit traits (fruit number, fruit diameter, fruit dry weight, anthocyanins content, polyphenols content).

	Leaf morphology				
	FA	SLA	LA	Leaf curve	FFW to FA ratio
P	**0.0178**	**0.012**	**0.0032**	**0.0801**	0.7366
Clone (P)	0.9642	0.9158	0.9726	0.1614	0.1142

	Crown				
	D	Twig length	Crown volume	Crown density	H
P	**0.0042**	**0.0317**	**0.0677**	0.1307	0.172
Clone (P)	0.3944	0.9888	0.9741	0.1092	0.1856

	**Fruit**				
	Fruit number	Fruit diameter	Fruit dry weight	Anthocyanin	Polyphenols
P	**0.0073**	**0.0095**	**0.0307**	0.3057	0.6908
Clone (P)	**0.023**	0.9486	**0.0621**	0.4328	0.1064

Significant effects (*P*-value < 0.1) are shown in bold type. The effects are provenance (Proven), Date and Clone.

### Variability among genotypes on fruit productivity and quality

Differences among provenances were found for fruit production but not for quality traits (Table 3). Plants from SanFer doubled the fruit number and dry weight of those coming from Enlagos (Fig. 5l,m). Fruit diameter was similar among the provenances, except for the 9% smaller berries from Enlagos (Fig. 5m). Among provenances, Mulchen seems to compensate low fruit production with a large fruit diameter (Fig. 5k,l).

**Figure 8:**
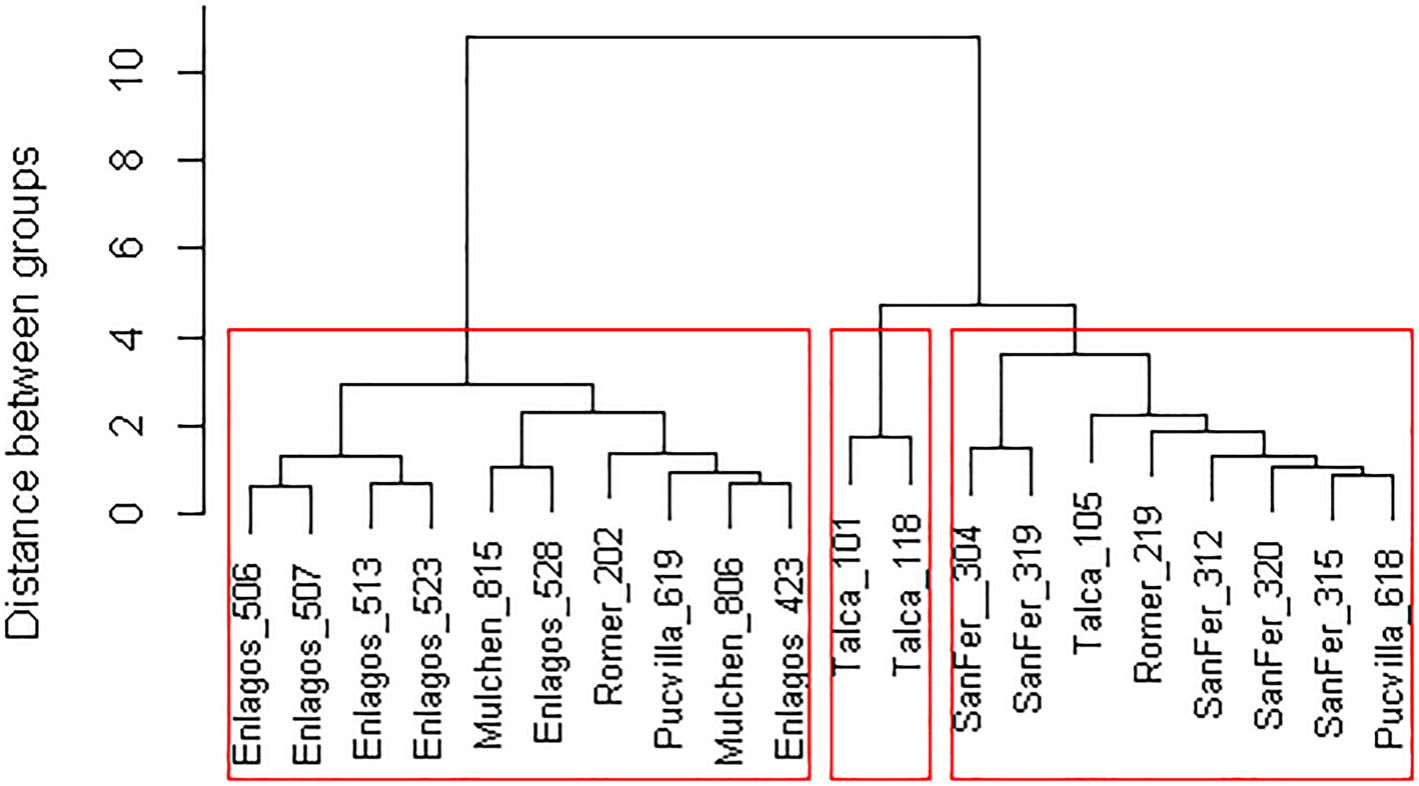
Cluster analysis based on fruit number (FFW), twig length, leaf area (FA) and intrinsic water use efficiency (WUEint) based in the scale distance.

Clonal variation within provenances was significant for fruit number and dry weight (Fig. 6b,c). One of the most productive clones (304) more than doubled fruit number and quadrupled dry weight compared with the less productive clone (528). Clonal differences were also detected by the cluster analysis (Fig. 8), with three main distinguished clusters. The first involved clones with high values for fruit production, twig growth, and leaf area but intermediate WUE_int_ performance. The second cluster with the local clones from Talca had a low fruit production and WUE_int_, but high twig growth and leaf area. The third cluster involved genotypes with low growth and fruit production but higher WUE_int_.

## Discussion

*Aristotelia chilensis* is distributed in a wide range of edaphoclimatic conditions in Chile and can be found from sclerophyllous to temperate rainforests. The reported genetic diversity of *A. chilensis* is high [cona y salgado], which is expected for species with anemophily and entomophile pollination. The study by Salgado et al.^32^ indicate little genetic structure but a relatively high gene flow between *A. chilensis* populations. Bastias et al.^40^ suggest a panmictic structure for A. chilensis sampled from 30° to 40° S, implying no mating restrictions for the species. This pattern of genetic structure is common in species with a large distribution range. *A. chilensis* is a dioecious species, whose berries are eaten by birds and the seed dispersed across the territory reducing the diversity among populations. According to the Köepen climate classification, the provenances assessed in this study ranged from the warm Mediterranean climate in the center part of the species distribution to the temperate coastal climate in the southern limit. From North to South, the provenance origin sites exhibit a decrease in mean temperature, solar irradiance, and altitude while considerably increasing precipitation and the De Martonne Index (Table 1). Overall, most of the phenotypic variation observed in this study in *A. chilensis* on fruit, leaf, and ecophysiological traits were more explained by differences between- than within-provenances. However, the variation among provenances was not clearly explained by a cline as expected.

Some studies comparing provenances in common garden experiments have shown better performance for local provenance origin than others more distant to the study site^23,34,41,42^, which is attributed to local adaptations (adaptative genetic changes) to specific edaphoclimatic conditions. However, other studies have shown a good performance of provenances out of their geographic origin, which are signs of maladaptive or non-adaptive genetic changes due to genetic drift or stresses induced by soil resource limitations^23,43^. Contrarily to our expectations, the provenance Talca, which is close to the common-garden experiment (and considered as a local provenance), had a moderate fruit production at expenses of a higher water loss (i.e., *g*_*s*_, *E*) (Fig. 5). Moreover, the high foliage development and low WUE_int_ do not support a local adaptation of this provenance to the warm-summer Mediterranean climate, characterized by water stress during the summer. Contrarily, a better performance was noted for the northern provenance SanFer, suggesting that the climate conditions at the study site were more favorable relative to their origin site and likely attributed to maladaptive or non-adaptive genetic changes, as has been mentioned by Gao et al.^43^. Overall, for most of the traits assessed, the differences among provenances were explained by the differences between northern provenances (mainly the SanFer provenance) and southernmost provenance Enlagos. The poor performance of provenance Enlagos suggests a local adaptation of this provenance to the temperate oceanic climate where it comes from.

### Genetic differentiation in leaf-physiological, morphological, and crown traits

Genetic differentiation was found for physiological, morphological, and crown traits. *A. chilensis* has one of the highest relative growth and photosynthetic capacity of the Chilean temperate rainy forest^44^ and even comparable to the values reported for some of the most productive sclerophyllous species in central Chile^45^. In this study, the values for the leaf-physiological parameters were in the range reported for the species^23,44,46,47^, although differences among provenances were found since the end of the ripening stage. All provenances experienced a higher variation *A*_*sat*_ within the maturation period, peaked at the end of the ripening period, and remained approximately steady during the rest of the growing period. In other species, this higher variation and increased photosynthetic activity within the ripening period has been related to a higher demand of photosynthates by fruits (i.e., sink), which drives physiological processes such as transpiration and gas exchange^48,49^. Thus, after berry ripening, this sink decreases the demand for photosynthates in *A. chilensis*, likely down-regulating the photosynthetic capacity to steady levels as has been reported in apple trees by Kelc et al. [^50^].

The high *g*_*s*_ and *E* observed in the local provenance Talca manifested in a significantly lower WUE_int_ compared to southern provenances (especially provenance Enlagos). Northern provenances came from sites with pronounced water restrictions compared to the southern provenances, so they were expected to be better adapted to the condition at the study site. However, specific adaptation to dry sites such as lower values for crown growth, gas exchange, foliar area, and leaf size were observed in the southernmost provenance Enlagos. The results of the provenance Talca disagree with the theory of specialization^51^ in which genotypes adapted to unfavorable conditions (i.e., a provenance originated in sites with high aridity) may have superior performance in restrictive environments but are unable to take advantage of favorable conditions (i.e., irrigation in the common-garden experiment), leading to low plasticity. Overall, the northernmost provenance SanFer seemed to take advantage of the climatic conditions at the study site maintaining higher productivity and WUE_int_ than the local provenance Talca, suggesting a high potential phenotypic plasticity for those provenances. Unlike the provenance Enlagos, which is likely well-adapted to wetter conditions, decreased physiological and morphological attributes when established in the drier conditions of the study site. This assertion is also supported by the lower leaf curvature ratio of this provenance. In this study, the leaf curvature ratio represents a measure of leaf rolling, which has been reported as a mechanism of avoidance of water loss^52^.

SLA did not increase with the latitude as has been found in other plant species^19,53^. The variation in SLA with the latitude is mainly associated with the gradient in light and temperature, but it may also be affected by other factors such as nutrient availability and water stress^54^. Interestingly, northern provenances marked the differences in SLA among provenances. SLA was the highest for provenances SanFer and Talca and lower for provenance Romer. In *A. chilensis*, a drier growth environment with more light availability tended to decreased SLA^47^, a potential local adaptation that was observed only in the provenance Romer. However, in other species, SLA tended to decrease with increasing precipitation at the site provenance origin, but it also showed to increase with water added by irrigation^19,22^, which likely applied to the results found for provenances SanFer and Talca.

Within provenances (i.e., clone level), the genetic differentiation was found significant in gas exchange parameters but not in the crown and leaf-morphological traits. Physiological traits may be more strongly affected by genetic than structural traits^22^, while the latter is mainly influenced by climate and nutrients^47,53^. Clones taken from a provenance develop under a similar climate, thus there is no climatic differentiation that could drive the genetic diversity on morphological traits. However, some provenances were not well-represented, which needs further research. Despite the low number of genotypes tested, there was a significant variation among clones in *A*_*sat*_, while the clonal performance on *g*_*s*_ and WUE_int_ was relatively stable within the growing period. In the end, E-BLUP of clones with higher and lower *g*_*s*_ differed 2-fold, while for WUE_int_ 3.7-fold, which offers the possibility to explore the selection of genotypes based on these traits.

### Genetic differentiation in ripening, berry production, and quality

Ripening lasted approximately 1 month, with significant differences in the ripening stage between provenances and clones throughout the dates. However, a clinal pattern was unclear, as the southernmost provenance Enlagos ripened fast even compared with the northernmost provenances, while the two clones from the mid-range of the distribution (e.g., provenance Mulchen) consistently had a delay in the ripening. Berry ripening is a complex process affected by soil resources, meso and micro-climate, and genetics^55^. It has been shown in other fruit tree species that only part of the genes influencing fruit maturation is affected by the environment and explains the varying responses of genotypes to specific environments^56^. Since the genotypes in our study grew in a common site, the results suggest a high genetic variation within and between provenances for berry ripening that overpasses a latitudinal cline. This high spontaneous genetic variation might explain the species’ success to prosper in different environmental conditions and agrees with the high genetic differentiation observed in genotypes with a common origin of other berries species. For instance, genetic differentiation in berry ripening was found between and within white and red grapevine cultivars in response to temperature^57,58^. Nesmith^59^ found high genotypic differentiation in southern-origin blueberry genotypes in the flowering time and fruit development in response to the chill hours at specific locations. Otherwise, Uleberg et al.^28^ mentioned potential local adaptations for bilberry clones (*Vaccinium myrtillus* L.), in which the berry ripening of northern origin genotypes (colder areas) was faster than the southern ones. In *A. chilensis* the genetic variation on berry ripening highlights the potential for genetic selection within the species to define early and late-ripening varieties.

Similar to other traits, the fruit size and yield differed mainly between the southernmost (EnLagos) and northern provenances, which suggest some local adaptation for the Enlagos provenance for these traits. There was also less variation in fruit production among clones within this provenance compared with the other provenances. Romero-Román et al.^60^ showed, for another native superfruit (*Berberis microphylla* G. Forst), that higher radiation tended to explain better the decrease in fruit size and weight than temperature. This might apply to our study, regarding that the gradient for the provenance origin was higher for global radiation than for the temperature. However, for *A. chilensis*, the decrease in the shortwave radiation (i.e., UV spectrum) might increase flower production and fruit set in some genotypes^29^. This partially explained our results because the PAR radiation is similar between the southernmost and northernmost provenance origin, whereas the UV radiation is higher in the southern provenance origin^61^. Moreover, in our experiment, plants of all provenances received the same amount of water by irrigation, which apparently was not enough for provenance EnLagos (adapted to high precipitation sites). This provenance had the lowest fruit traits (i.e., fruit number, diameter, and dry weight) and did not privilege reproductive over vegetative growth. It is known that water irrigation strategies that diminish water usually reduces vegetative growth promoting reproductive growth, i.e., number of fruits^62,63^, but this was not the case for EnLagos plants and might be explained by the high plasticity of this provenance, climate differences, or both. Thus, it might be possible that the higher radiation of the common-garden experiment dampened reproductive growth of the EnLagos provenance, particularly clone 528. However, this hypothesis needs further research.

Although genotypic differences in the fruit productivity were expressed in the common-garden experiment, these were not significant for the anthocyanin and polyphenol content. The accumulation of these secondary compounds depends on the fruit ripening stage, genetic, and environmental factors such as temperature, water stress, irradiation, soils, and altitude^28,64–67^. Apparently, none of the above-mentioned factors affected the berry quality in the genotypes under study. It is known that an excess or deficit of water influence the acidity of berry crops^68^, but results depend on the species, the intensity of the water stress applied, and the maturity stage. In apples, bioactive compounds such as anthocyanins tended to decreased with higher temperatures and increased with solar radiation while also differing between accessions^69^. In the present study, the higher temperatures and radiation experienced by the southern provenances at the experimental site were not likely sufficient to drive changes or local adaptations in the biosynthesis of these secondary compounds. According to Gonzalez-Villagra et al.^70^, in *A. chilensis*, severe water stress may increase the leaf anthocyanins and polyphenols content. In grapes, limited irrigation increased the berries anthocyanin concentration, the galloydation of seed, and skin proanthocyanidins, all related to greater wine properties and antioxidant activity^71^. In our study, the irrigation was provided continuously by a drip-irrigation system, alleviating any potential water stress on plants and the differences among genotypes in secondary compounds. In the case of *A. chilensis*, the pedoclimatic conditions likely excerpt little influence on anthocyanin content, as has been found in blueberry cultivars by Spinardi et al.^65^.

There is a need to select better genotypes and cultivated under intensive management practices to respond to the future demand for berries of *A. chilensis*. The domestication of the species might reduce the anthropogenic pressure on natural populations. Our common-garden experiment represents the standard management practices currently implemented in other fruit trees in the Mediterranean zone of Chile (including irrigation) and likely the most productive age. The combined analysis of traits by the clustering analysis suggests the potential genetic variation of the species that can guide the domestication process. Moreover, although three clusters were defined and related to latitude, there are some exceptions (i.e., clone 202 and 618). The genetic variation in this study offers the possibility of defining ideotypes for specific sites (e.g., Yáñez et al.^72^ in Loblolly pine trees). Then, further research is needed on multi-site trials to estimate clonal genetic parameters.

Regarding the broad distribution of *A. chilensis*, this study suggests that the species has a great phenotypic variation, as southern provenances (except the southernmost) had a good performance in the climatic conditions of the study site, compared to the northern provenances in some traits. However, the southernmost provenance differed in most of the traits compared with the other provenances, which suggests a local adaptation to wetter sites and with the consequent decrease in performance in drier climates. Regarding the latitudinal gradient in most climatic variables, the variation among provenances was not clearly explained by a cline. Genetic differentiation was higher between than within provenances. Overall, differences among provenances were evident for leaf-physiological and morphological, fruit, and crown traits, whereas at clone level, the differences were expressed only for physiological and fruit traits. The higher phenotypic variation of the provenances understudy suggests a great potential for selection within the species and resilience under the expected changes in the environment due to climate change.
